# Genome-Wide Identification of Long Noncoding RNAs and Their Responses to Salt Stress in Two Closely Related Poplars

**DOI:** 10.3389/fgene.2019.00777

**Published:** 2019-09-05

**Authors:** Jianchao Ma, Xiaotao Bai, Wenchun Luo, Yannan Feng, Xuemin Shao, Qiuxian Bai, Shujiao Sun, Qiming Long, Dongshi Wan

**Affiliations:** ^1^State Key Laboratory of Grassland Agro-Ecosystem, School of Life Sciences, Lanzhou University, Lanzhou, China; ^2^Key Laboratory of Plant Stress Biology, State Key Laboratory of Cotton Biology, School of Life Sciences, Henan University, Kaifeng, China

**Keywords:** long noncoding RNAs, poplars, tissue-specific expression, plant growth, salt response

## Abstract

Long noncoding RNAs (lncRNAs) are involved in various biological regulatory processes, but their roles in plants resistance to salt stress remain largely unknown. To systematically explore the characteristics of lncRNAs and their roles in plant salt responses, we conducted strand-specific RNA-sequencing of four tissue types with salt treatments in two closely related poplars (*Populus euphratica* and *Populus alba* var. *pyramidalis*), and a total of 10,646 and 10,531 lncRNAs were identified, respectively. These lncRNAs showed significantly lower values in terms of length, expression, and expression correction than with mRNA. We further found that about 40% and 60% of these identified lncRNAs responded to salt stress with tissue-specific expression patterns across the two poplars. Furthermore, lncRNAs showed weak evolutionary conservation in sequences and exhibited diverse regulatory styles; in particular, tissue- and species-specific responses to salt stress varied greatly in two poplars, for example, 322 lncRNAs were found highly expressed in *P. euphratica* but not in *P. alba* var. *pyramidalis* and 3,425 lncRNAs were identified to be species-specific in *P. euphratica* in response to salt stress. Moreover, tissue-specific expression of lncRNAs in two poplars were identified with predicted target genes included *Aux/IAA*, *NAC*, *MYB*, involved in regulating plant growth and the plant stress response. Taken together, the systematic analysis of lncRNAs between sister species enhances our understanding of the characteristics of lncRNAs and their roles in plant growth and salt response.

## Introduction

Salinity is one of the most important environmental factors limiting plant growth and development and results in crop loss in semiarid and arid areas (Boyer, 1982). The molecular mechanisms underlying the response to salt stress in plants have been well characterized, and most studies have mainly focused on the functional study of protein-coding genes, such as *HKT1*, Na^+^/H^+^ exchanger (*NHX*), and *SALT OVERLY SENSITIVE* (*SOS*, also known as *NHX7*) (Shi et al., 2002; Venema et al., 2003; Byrt et al., 2007). In the past decade, an increasing number of noncoding RNAs (ncRNA) have been identified to function as precursors of microRNAs (miRNAs) and other small RNAs or as miRNA target mimics, and involved in plant response to salt stress. For example, 7,361 and 7,874 long ncRNAs (lncRNAs) were identified from salt stress-treated the leaf and root of *Medicago truncatula* (Wang et al., 2015). Salt stress can also alter the accumulation of lncRNAs in *Arabidopsis* (Ben Amor et al., 2009). However, the regulatory mechanism of lncRNAs underlying the response to salt stress remains largely unknown.

ncRNAs are a set of RNAs that have no capacity to code for proteins. They used to be considered inconsequential transcriptional “noise” because of the limited amount of information regarding their functions (Ponjavic et al., 2007; Struhl, 2007). However, recently, many studies have shown that ncRNAs play important regulatory roles in a wide range of biological processes (Wilusz et al., 2009; Kim et al., 2011). In general, based on their sequence lengths, ncRNAs are divided into small RNAs and lncRNAs. Small RNAs can be further grouped into miRNAs and small interfering RNAs (siRNAs) (Brosnan and Voinnet, 2009), which are less than 50 nucleotides in length. On the other hand, lncRNAs are defined as a group of ncRNAs that are more than 200 nucleotides in length (Rinn and Chang, 2012). Unlike mRNAs, the expression of lncRNA is usually exhibited in a tissue- and cell-specific manner, at low levels, and with the transcripts being localized to subcellular compartments (Wilusz et al., 2009; Cabili et al., 2011). On the basis of their genomic localizations with respect to protein-coding genes, lncRNAs can be classified as long noncoding natural antisense transcripts (lncNATs), long intergenic ncRNAs (lincRNAs), long intronic noncoding RNAs and overlapping lncRNAs, which partially overlap with protein-coding genes (Bazin et al., 2017). lncRNAs can affect gene expression by binding specific regions in the target genes and cooperating with proteins or transcriptional elements to regulate transcription. There are two models for how lncRNAs regulate gene expression: those acting in close proximity (acting in *cis*) and those acting at a distance (acting in *trans*) to their position in the genome (Ponting et al., 2009). The varied regulation styles of lncRNAs depend not only on their specific structures and sequences but also on their binding to transcriptional elements (Quan et al., 2015). Contrary to protein-coding genes, most lncRNAs lack strong conservation of nucleotide sequences among species (Necsulea et al., 2014).

In recent years, numerous studies have shown that ncRNAs act as regulatory molecules in various developmental processes and respond to biotic or abiotic stress in plants (Zhang and Chen, 2013; Liu et al., 2015a) and are thus considered to be potential regulators of plant responses to the environment. For example, plant lncRNAs have been found to be involved in numerous biological regulatory processes including gene silencing (Franco-Zorrilla et al., 2007; Wu et al., 2013), flowering time (Liu et al., 2010; Heo and Sung, 2011; Wang et al., 2014b), fruit development and ripening (Tang et al., 2016), responses to biotic and abiotic stress (Ben Amor et al., 2009; Zhu et al., 2014; Cui et al., 2017), wood formation (Chen et al., 2015), the secondary growth of plants (Zhou et al., 2017), and other important developmental pathways. Among them, lncRNA can not only regulate gene transcription and epigenetics in the nucleus (Gosai et al., 2015; Bazin et al., 2017) but is also associated with mRNA stability and translation in the cytoplasm (Gong and Maquat, 2011). However, the regulatory function of the majority of lncRNAs in plants remains largely unknown. Therefore, systematic identification of lncRNAs with specific function that were involved in plant adaptation, diversity, and even speciation is necessary.

*Populus euphratica* Oliv is a well-known halophyte tree, which is distributed mainly in arid or semi-arid regions of western China and central and western Asia. *P. euphratica* trees have a high tolerance to salt and drought stress and are a model tree for studying salt tolerance in plants (Ma et al., 2013; Ma et al., 2018b). *P. alba* var. *pyramidalis* is a variety of P. alba and well known for its fast growth (Yang et al., 1992). Both poplars have diversified recently and are closely related species phylogenetically, *P. alba* var. *pyramidal*is exhibits a salt sensitive phenotype compared with *P. euphratica*. Therefore, a comparative analysis of the expression patterns from salt treatment between the two closely related poplar species will contribute to deciphering the regulatory pathways that respond to salt stress. Here, we systemically identified and characterized lncRNAs from four tissue types (leaf, phloem, xylem, and root). Further analysis aimed to explore the conservation of sequences and expression patterns of lncRNAs between two closely related species as well as the roles of lncRNAs in plant growth and responses to salt stress in the two poplars. The comparison will allow us to better understand the characteristics of lncRNAs and will provide insights into the roles of lncRNAs in the salt response and plant growth.

## Materials and Methods

### Plant Materials

*P. alba* var. *pyramidalis* and *P. euphratica* saplings (2 years old) were collected and grown in a greenhouse with a photoperiod of 16 h light/8 h darkness (6:30-22:30) and 60% humidity at 25°C. The saplings were treated for 7 days with a solution containing 0, 150, or 300 mM NaCl, of them, 0 mM NaCl solution treatment was the control. Three replicates from three individual saplings were treated with the same salt concentration. The treatment has been descripted in Yu et al. (2017), and the leaf, phloem, xylem, and root tissues were collected from similar stages at 14:00 to 15:00 for RNA sequencing (RNA-Seq). For K^+^ and Na^+^ contents measurement, the tissues from leaf and root, respectively, were collected from similar stages and dried at 65°C for 2 days. Dried tissues (0.1 g) were extracted with 10 ml, 0.1 M HNO_3_ for 2 h, After filtering by 0.45 μm filter membranes, the contents of K^+^ and Na^+^ were determined using Inductively Coupled Plasma Optical Emission Spectrometer (ICP-OES) (Optima 4300DV/5300DV; Perkin-Elmer) as described in Baxter et al. (2010).

### RNA Sequencing

Total RNA was extracted from four tissue types (leaf, phloem, xylem, and root) from each sample for RNA sequencing using a CTAB procedure (Porebski et al., 1997). Each sample was performed in triplicate using three individual saplings treated under the same conditions. A total of 36 samples were used for the subsequent experiments with RNA integrity number (RIN) values over 8.0 for each poplar. Whole-transcriptome libraries were constructed, and deep sequencing was performed by the Annoroad Gene Technology Corporation (Beijing, China). Whole-transcriptome libraries were constructed using NEB Next Ultra Directional RNA Library Prep Kit for Illumina (NEB, Ispawich, USA) according to the manufacturer’s instructions. Libraries were controlled for quality and quantified using the BioAnalyzer 2100 system and qPCR (Kapa Biosystems, Woburn, MA, USA). To identify antisense transcripts, a strand-specific RNA-seq strategy was adopted, and RNA-seq libraries were generated using the SOLiD™ Whole Transcriptome Analysis Kit (ABI). The resulting libraries were initially sequenced on a HiSeq 2500 instrument that generated paired-end reads of 125 nucleotides. All sequencing data have been submitted to the NCBI Sequence Read Archive (SRA accession numbers SRX3504248-SRX3504283).

### Prediction of lncRNAs and Identification of Salt-Response lncRNAs

The quality of the paired-end RNA-seq reads was determined using FASTX-Toolkit version 0.0.13 (http://hannonlab.cshl.edu/fastx_toolkit/index.html) with default parameters through removing low-quality reads, adaptor sequences, and sequences shorter than 20 nucleotides. The clean reads were aligned to the *P. alba* var. *pyramidalis* (http://bigd.big.ac.cn/gwh) (Ma et al., 2018a) and *P. euphratica* genomes (Ma et al., 2013) using Tophat (Trapnell et al., 2014), allowing for three base mismatches. Reads with no more than three mismatches were used to separately assemble the transcripts of each sample using Cufflinks (Trapnell et al., 2014) and based on the two-reference genomes. Expression levels of the assembled transcripts were calculated and normalized using fragments per kilobase of transcript per million fragments (FPKM) by Cufflinks (Trapnell et al., 2014). The prediction of lncRNAs from RNA-seq data was performed according to Sun et al. (2012), and the pipeline is shown in **Supplementary Figure S1**. Transcripts with FPKM <1 in each sample were removed. Any transcripts that were shorter than 200 bp were discarded. The coding potential of the remaining transcripts was evaluated using coding potential calculator (CPC) software (http://cpc.cbi.pku.edu.cn/) (Kong et al., 2007) and Coding Noncoding Index (CNCI) software (https://github.com/www-bioinfo-org/CNCI) (Sun et al., 2013). When using CPC, we used the NCBI protein data base as a reference. All transcripts with CPC scores >0 or a CNCI >0 were discarded. The lncRNAs were classified into intergenic, intronic, antisense, and sense lncRNAs using the cuffcompare program in the Cufflinks suite (Roberts et al., 2011; Trapnell et al., 2014). The change of lncRNA expression was calculated as the fold change (FC) in two samples (0 vs 150, 0 vs 300 and 150 vs 300 mM NaCl) in each tissue. Only the lncRNAs that met the criteria of log_2_ FC ≥1 or ≤−1 with *P* values < 0.05 were considered to be salt responsive.

### Prediction of Target Gene

The potential target genes of salt-responsive lncRNAs were predicted according to their regulatory effects, which were divided into *cis*- and *trans*-acting. Two independent algorithms were used. The first algorithm searched for potential *cis* target genes that are physically close to lncRNAs (within 10 kb) by using genome annotation. The genes transcribed within a 10-kb window upstream or downstream of lncRNAs were considered to be potential *cis* target genes (Jia et al., 2010; Tian et al., 2016). The criteria used for the prediction of potential *cis* targets are described in Jia et al. (2010). The second algorithm searched for potential *trans* targets in the Populus mRNA database and is based on mRNA sequence complementarity and RNA duplex energy prediction, assessing the impact of lncRNA binding on complete mRNA molecules. First, we used BLAST to select target sequences that were complementary to the lncRNA, setting the E value at <1e^−5^ and identity at ≥95%. Then, RNAplex software was used to calculate the complementary energy between two sequences for further screening and to select potential *trans*-acting target genes (RNAplex −e^−60^) (Tafer and Hofacker, 2008).

### Conserved Elements and Specific Expression of *Populus* lncRNAs

The expression of RNAs was regulated by RNA-binding proteins, motifs or elements were the regions recognized by these RNA-binding proteins (Ray et al., 2013). These motifs displayed deep evolutionary conservation and were associated with distinct functional role (Wuchty et al., 2003). Conserved elements in lncRNAs were identified using DREME online software specially designed to find relatively short motifs with E values < 0.05 (Bailey, 2011). The tissue specificity of lncRNA expression was evaluated according to the tissue-specific index, which ranges from 0 for housekeeping genes to 1 for tissue-restricted genes, as described by Yanai et al. (2005). The index was calculated as: tissue-specific $\mathrm{index}=\frac{\sum_{i=1}^{n}\left(1-\frac{\mathrm{Exp}\;i}{\mathrm{Exp}\;\max}\right)}{n-1}$, where n is the number of tissues; Exp *i* is the expression value of each lncRNA in the tissue, *i*; and Exp *max* is the maximum expression value of each lncRNA among all tissues. Only the lncRNAs showing a tissue-specific index > 0.9 were considered to be tissue-specific. The reliability of the RNA-Seq analyses has been verified by quantitative real-time PCR analysis in previous and current studies (Yu et al., 2017). An 18S RNA was used for the internal reference gene to normalize the relative expression levels of the lncRNAs.

### Gene Ontology (GO) Enrichment Analysis and Identification of Homologous lncRNAs

Before GO and pathway enrichment analysis, the predicted target genes were annotated by Blast2GO (Conesa et al., 2005). Then, GO terms were identified using suggested backgrounds, and the *p*-value value cutoff was set as 0.05. Homologous lncRNAs were identified using the BLAST method. Only the BLAST results of individual lncRNAs to themselves were identified as being homologous.

## Results

### Salt Treatment of the Two Poplars and Identification of lncRNAs

**Figure 1:**
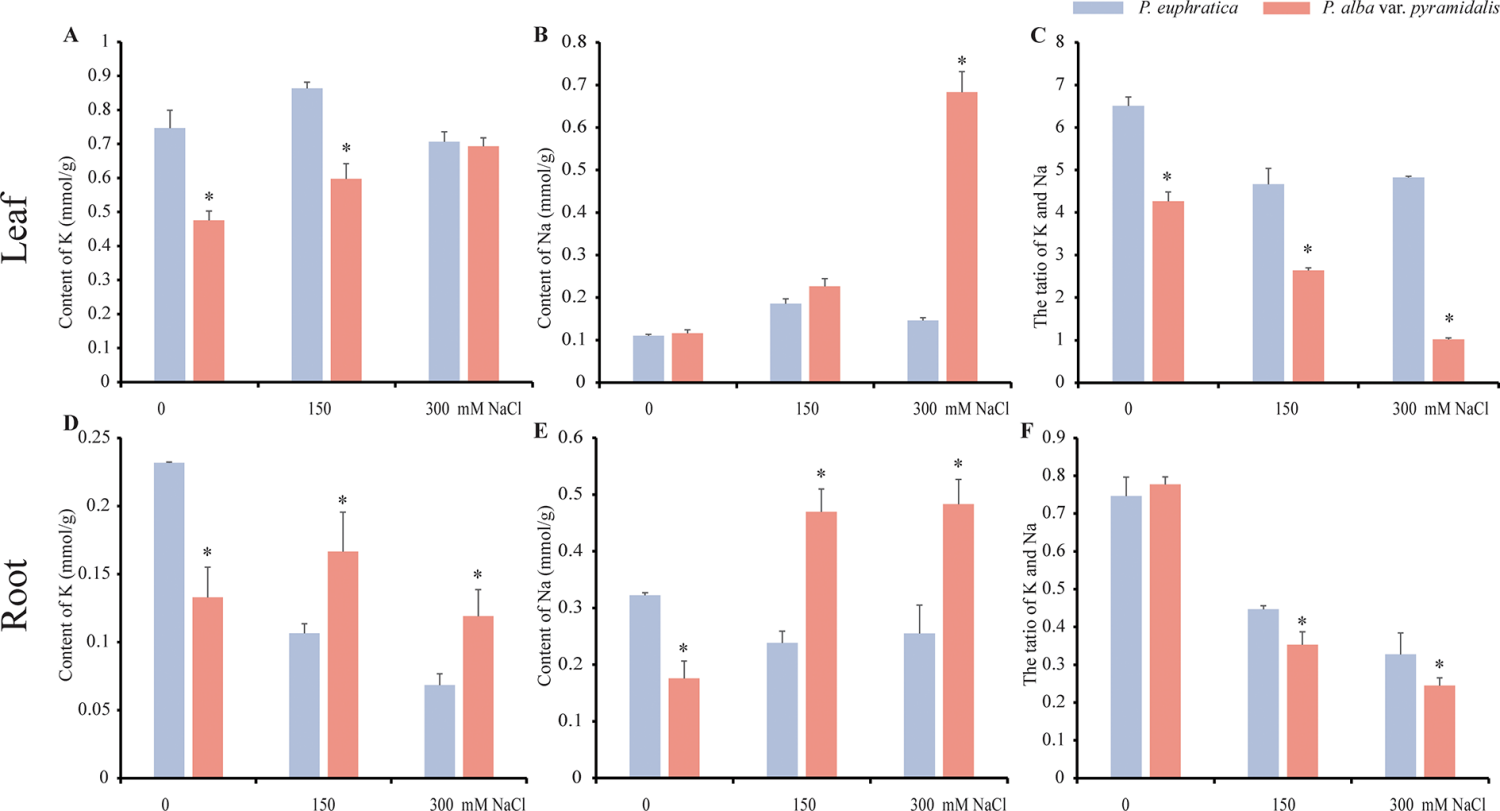
The contents of Na^+^ and K^+^ in the leaf and root under different salt concentrations in *P. euphratica* and *P. alba* var. *pyramidalis*. **(A)** The contents of K^+^ in the leaf under different salt concentrations. **(B)** The contents of Na^+^ in the leaf under different salt concentrations. **(C)** The ratio of K^+^/Na^+^ in the leaf under different salt concentrations. **(D)** The contents of K^+^ in the root under different salt concentrations. **(E)** The contents of Na^+^ in the root under different salt concentrations. **(F)** The ratio of K^+^/Na^+^ in the root under different salt concentrations. * indicated significance difference between the two poplars.

To examine the effects of salt on the growth of *P. alba* var. *pyramidalis* and *P. euphratica*, the two poplars saplings were treated with NaCl at different concentrations (0, 150, and 300 mM). We found that *P. alba* var. *pyramidalis* (10 of all 10 saplings) showed an obvious phenotype of losing water after 7 days of 300 mM NaCl treatment, whereas *P. euphratica* (9 of all 9 saplings) had no phenotype, confirming that *P. euphratica* has more tolerance to salt stress than *P. alba* var. *pyramidalis*. Besides, more K^+^ contents in root and leaf, more Na^+^ contents in root were found in *P. euphratica* under 0 mM NaCl treatment (**Figure 1**), indicating that *P. euphratica* could hold more K^+^ and Na^+^ compared with *P. alba* var. *pyramidalis*. We further examined the K^+^ and Na^+^ contents in the leaves and roots to estimate the accumulation of and transportation of ions when treated with salt. The results showed that the K^+^ and Na^+^ contents elevated at 0 and 150 mM NaCl treatment but decreased at 300 mM NaCl treatment in the leaves of *P. euphratica* (**Figures 1A, B**), whereas both increased greatly in the leaves of *P. alba* var. *pyramidalis* (**Figures 1A, B**). In the roots, the K^+^ content decreased, the Na^+^ content decreased at 0 and 150 mM NaCl treatment in the *P. euphratica* samples (**Figures 1D, E**), and the Na^+^ content increased significantly in *P. alba* var. *pyramidalis* (**Figure 1E**). As a result, the ratio of K^+^/Na^+^ decreased in the leaves and roots in both poplars under salt stress with the ratio in *P. alba* var. *pyramidalis* decreasing more significantly than that in *P. euphratica*, indicating a different response level or salt response mechanism in the two poplars (**Figures 1C, F**).

**Figure 2:**
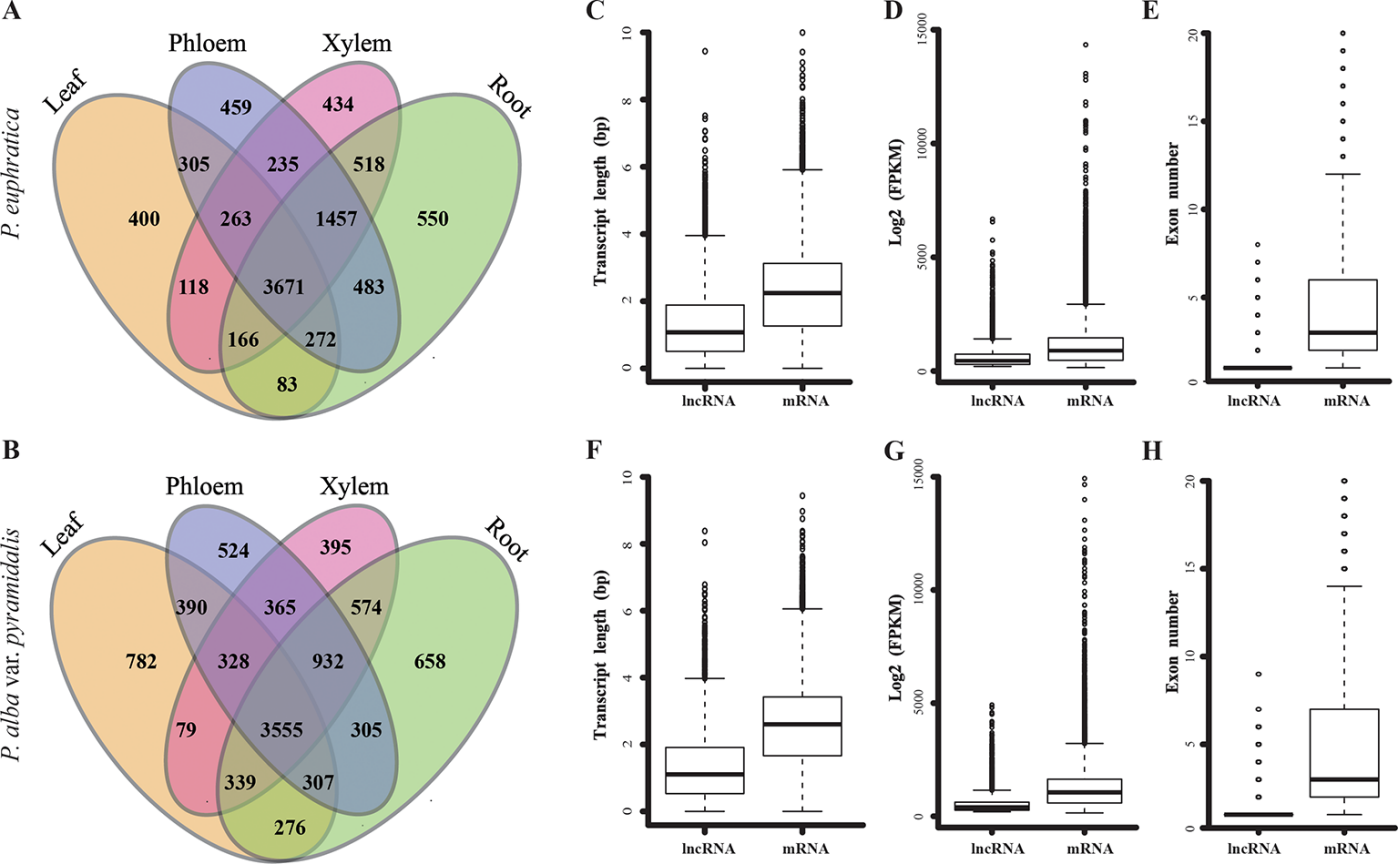
Identification and characteristics of lncRNAs in two species of poplar. **(A**–**B)** Distribution of identified lncRNAs in the four tissue types of the two poplars. **(C**–**E)** Boxplot of the transcript length, exon number, and FPKM value of lncRNA and mRNA in *P. euphratica*. **(F**–**H)** Boxplot of the transcript length, exon number and FPKM value of lncRNA and mRNA in *P. alba* var. *pyramidalis*.

Based on the salt treatment, the four tissues from two poplars saplings were used to perform high-throughput RNA-seq (stand-specific) and identify lncRNAs in a systematic genome-wide. In total, we identified 10,646 and 10,531 lncRNAs with FPKM >1 in at least one library of *P. euphratica* and *P. alba* var. *pyramidalis*, respectively (**Figures 2A, B**; **Supplementary Data S1-3**). These lncRNAs were further classified into 4,423 long intronic noncoding RNAs, 1,014 overlapping lncRNAs, 4,761 lincRNAs and 448 lncNATs in *P. euphratica*, and 4,221 long intronic noncoding RNAs, 1,184 overlapping lncRNAs, 4,615 lincRNAs, and 394 lncNATs in *P. alba* var. *pyramidalis*, with most lncRNAs being classified as long intronic noncoding RNAs or overlapping lncRNAs that partially overlap with protein-coding genes. There were 3,671 and 3,555 lncRNAs expressed in all four tissue types of *P. euphratica* and *P. alba* var. *pyramidalis*, respectively, and at least 400 lncRNAs were unique among the four tissue types of the both poplars (**Figures 2A, B**). We further found that the lengths, expression levels, and exon numbers of the lncRNAs were all shorter or lower than those of the mRNAs of both poplars (**Figures 2C–H**). Additionally, clustering analysis suggested that the lncRNAs displayed a low relationship between the four tissue types compared with mRNAs (**Supplementary Figure S2**), indicating a tissue-specific expression pattern.

### Tissue-Specific Expression of lncRNAs and Their Putative Roles in Plant Growth

**Figure 3:**
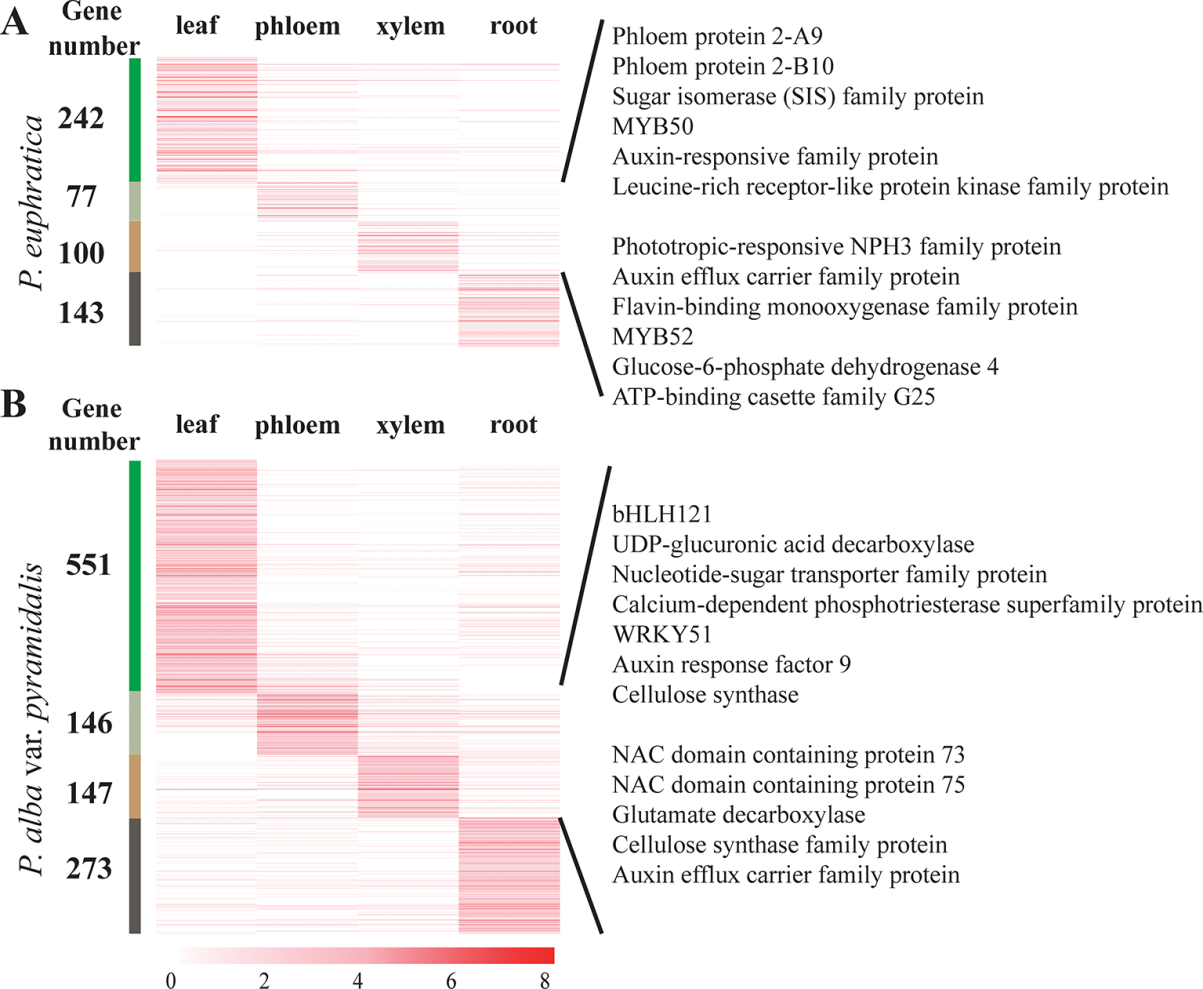
Tissue-specific expression of lncRNAs and their roles in plant growth. **(A)** Tissue-specific expression of lncRNAs and their roles in plant growth in phloem and xylem in *P. euphratica*. **(B)** Tissue-specific expression of lncRNAs and their roles in plant growth in phloem and xylem in *P. alba* var. *pyramidalis*. LncRNAs specifically expressed in different tissues were clustered together and marked on the left with different colors. The number on the left means the number of tissue-specific expressed lncRNAs.

The lncRNA expression profiles of both poplar species in terms of the four tissue types and salt stress conditions were studied. We found that most lncRNAs were expressed in more than one tissue, whereas 562 and 1,117 lncRNAs showed tissue-specific expression in *P. euphratica* and *P. alba* var. *pyramidalis* using the tissue-specific expression index (**Figures 3A, B**). Nearly half of these lncRNAs were preferentially expressed in the leaf. Classification analysis indicated that about half (47.5%) of these tissue-specific lncRNAs belonged to the lincRNAs in the two poplars. To explore whether these lncRNAs had conserved elements, 9 and 27 conserved elements were identified among these tissue-specific lncRNAs in *P. euphratica* and *P. alba* var. *pyramidalis*, respectively (**Supplementary Data S4**).

A previous study indicated that lncRNA can regulate plant growth by regulating the Aux/IAA gene family (Liscum and Reed, 2002). We found that there were 29 lncRNAs regulating the expression of Aux/IAA gene family members in both poplars. Additionally, specifically expressed lncRNAs in phloem or xylem were found to be involved in “cellulose synthase” and “auxin response” and were predicted to regulate plant growth transcriptional factors, such as *WRKY*, *NAC*, and *MYB* to promote plant growth (Eulgem et al., 2000; Cassan-Wang et al., 2013; Jervis et al., 2015) (**Figures 3A, B**).

### Differentially Expressed (DE) lncRNAs Under Salt Stress

**Figure 4:**
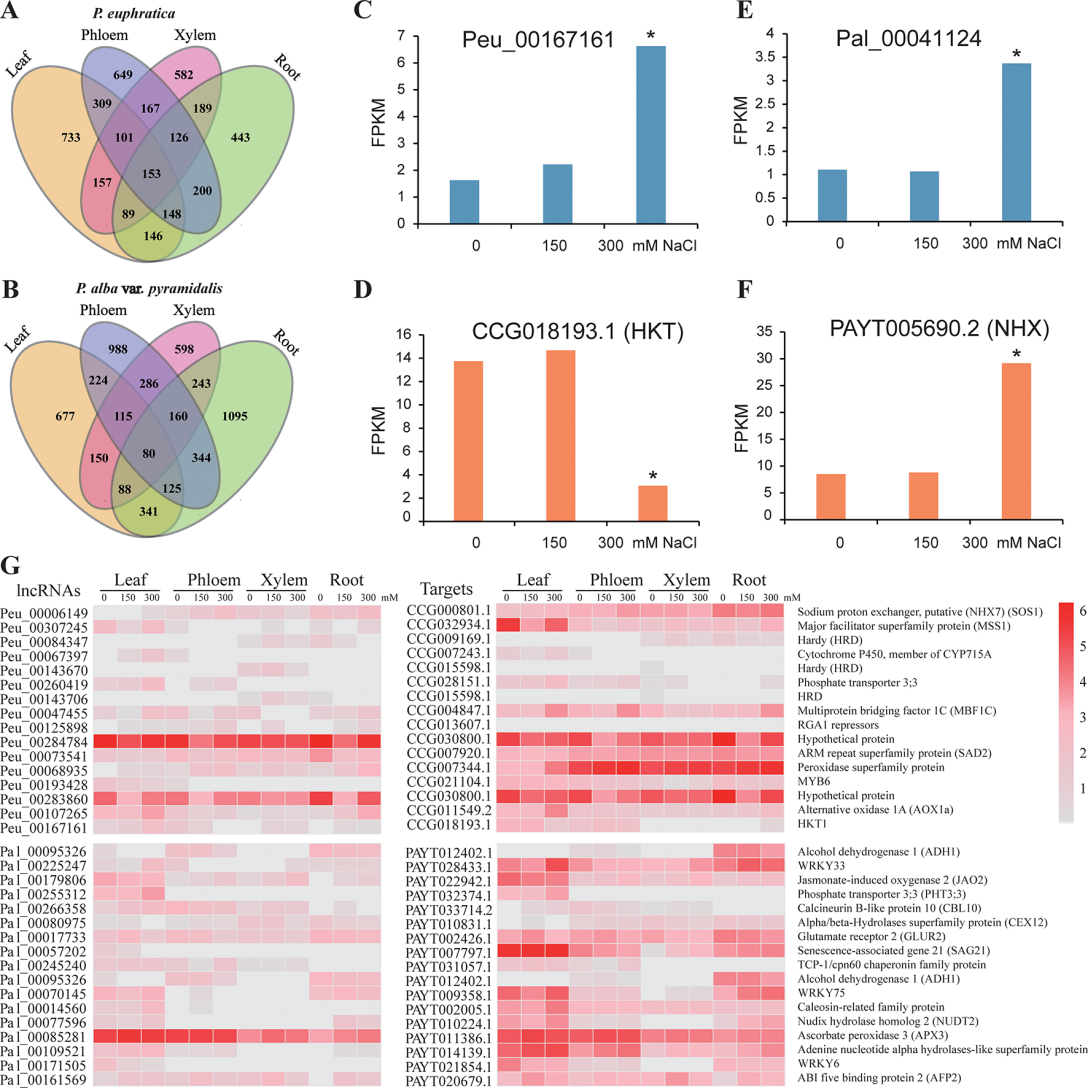
LncRNAs in response to salt stress. **(A**–**B)** Distribution of DE lncRNAs in the four tissue types under salt stress in the two poplars. **(C**–**D)** Expression conditions of one lncRNA and its potential target gene *HKT1* under salt stress. **(E**–**F)** Expression conditions of one lncRNA and its potential target gene *NHX* under salt stress. **(G)** Examples of lncRNAs response to salt stress in *P. euphratica* and *P. alba* var. *pyramidalis*. * indicated significance difference expressed under 300 mM NaCl treatment.

We identified 4,199 (39.4% of the total; 5.3% belonging to lncNATs; 49.2% belonging to lincRNAs; 36.7% belonging to long intronic noncoding RNAs, 8.8% belonging to overlapping lncRNAs), and 6,048 (60.8% of total; 4.8% belonging to lncNATs; 45.7% belonging to lincRNAs; 38.8% belonging to long intronic noncoding RNAs; 10.8% belonging to overlapping lncRNAs) lncRNAs that responded to salt stress in *P. euphratica* and *P. alba* var. *pyramidalis*, respectively (**Figures 4A, B**, **Supplementary Data S5–6**). 38 and 53 conserved elements were identified in these DE lncRNAs in two poplars (**Supplementary Data S4**). Four of these DE lncRNAs were further confirmed by qRT-PCR analysis (**Supplementary Figure S3**). Because lncRNAs play important roles in regulating gene expression, identification and analysis of their target genes may help us to explore their potential functions. Computational prediction identified a set of 6,840 and 9,838 potential target genes (PTGs), including 8,171 and 12,361 lncRNA-target pairs, for these DE lncRNAs in *P. euphratica* and *P. alba* var. *pyramidalis*, respectively (**Supplementary Data S7–8**). We then analyzed the relationship between the expression of the lncRNAs and the PTGs under salt stress among the four tissue types. Only approximately 5% of the lncRNA-target pairs showed the same or opposite expression trends among the four tissue types and under different salt concentrations in both poplars (**Supplementary Figure S4**).

Further functional enrichment analysis suggested that these PTGs were representatively enriched in the “intrinsic and integral component of membrane,” “transcription factor complex,” and “oxidoreductase activity” categories. Additionally, there were 367 and 481 PTGs belonging to ion transporter proteins in the two poplars, including the *HKT1* and *NHX* genes, which play important roles in the balancing of the Na^+^ and K^+^ contents. For instance, the potential target *HKT1* gene located downstream of *Peu_00167161* showed opposite expression patterns, and *Pal_00041124* located in the intron of *NHX* gene showed similar expression patterns in the leaf (**Figures 4C–G**; **Supplementary Figure S3A**). There were 598 and 771 PTGs belonging to the category of transcriptional factors, which also play important roles in salt resistance. For example, *Pal_00225247* showed similar expression patterns with its potential target salt response factor *PalWRKY33* in the leaf (**Supplementary Figure S3B**) and xylem and showed opposite expression patterns with *WRKY33* in phloem (**Figure 4G**) (Zhou et al., 2015). We also identified target genes related to oxidoreductase activity and osmotic balance (**Figure 4G**). All these results indicate that lncRNAs in the two species of poplar are involved in the response to salt stress not only by regulating structural proteins related to ion homeostasis and transportation, such as HKT family and NHX family proteins, but also by regulating the expression of transcription factors.

### lncRNA Responses to Stress by Different Salt Concentrations

**Figure 5:**
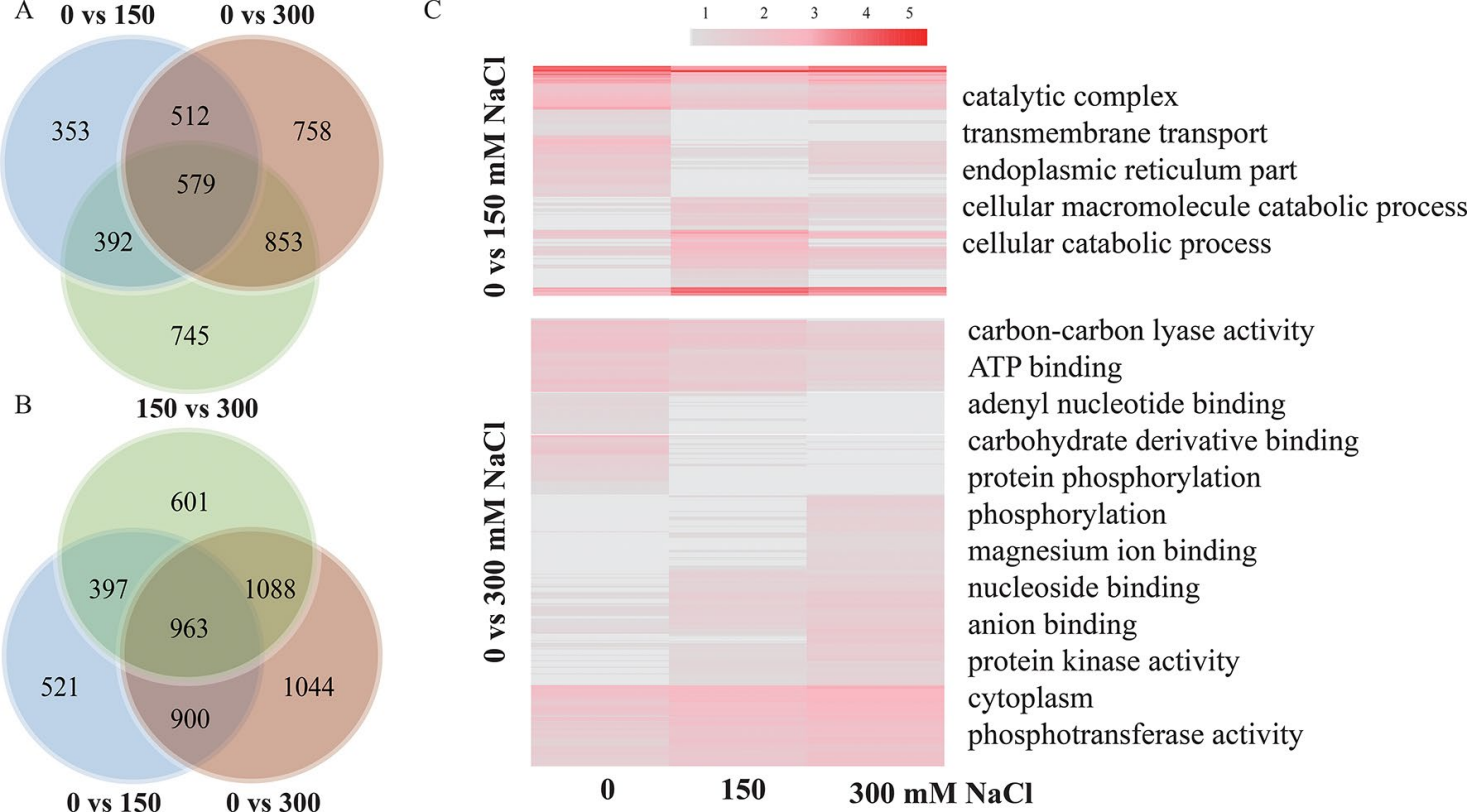
LncRNAs response to different salt concentrations. **(A)** lncRNAs response to different salt concentrations in *P. euphratica*. **(B)** lncRNAs response to different salt concentrations in *P. alba* var. *pyramidalis*. **(C)** GO enrichment of lncRNAs responses to different salt concentrations in *P. euphratica*.

A total of 1,836, 2,702, and 2,569 lncRNAs were differentially expressed (log_2_ FC >1 or < −1 and *P* < 0.05) between samples treated with 0 and 150 mM, 0 and 300 mM, and 150 and 300 mM NaCl in *P. euphratica* (**Figure 5A**; **Supplementary Data S9–10**), whereas the numbers in *P. alba* var. *pyramidalis* were 2,781, 3,995, and 3,049, respectively (**Figure 5B**). We found that lncRNA was primarily differentially expressed under high salt concentrations, and functional annotation indicated that DE lncRNAs under different salt concentrations showed different functions. As shown in **Figure 5C**, using *P. euphratica* as an example, DE lncRNAs between 0 and 150 mM NaCl were mainly enriched in “catalytic complex” and “transmembrane transport,” whereas DE lncRNAs between 0 and 300 mM NaCl were mainly enriched in “ATP binding,” “adenyl nucleotide binding,” and “protein phosphorylation.” In addition, the response to salt stress of lncRNAs and PTGs also varied under different salt concentrations. For example, we found that at concentrations under 150 mM NaCl, *Peu_00073541* with its *trans* target *SAD2*, which is involved in ABA signaling and the drought response in the root, differently expressed (Verslues et al., 2006), while most other lncRNAs and their potential target genes, such as *NHX7*, *INT1*, and *HRD* (Sakamoto et al., 2008; Abogadallah et al., 2011; Pehlivan et al., 2016) only differently expressed at 300 mM NaCl treatment, indicating specific responses to different salt concentrations.

### Tissue-Specific DE lncRNAs Under Salt Stress

**Figure 6:**
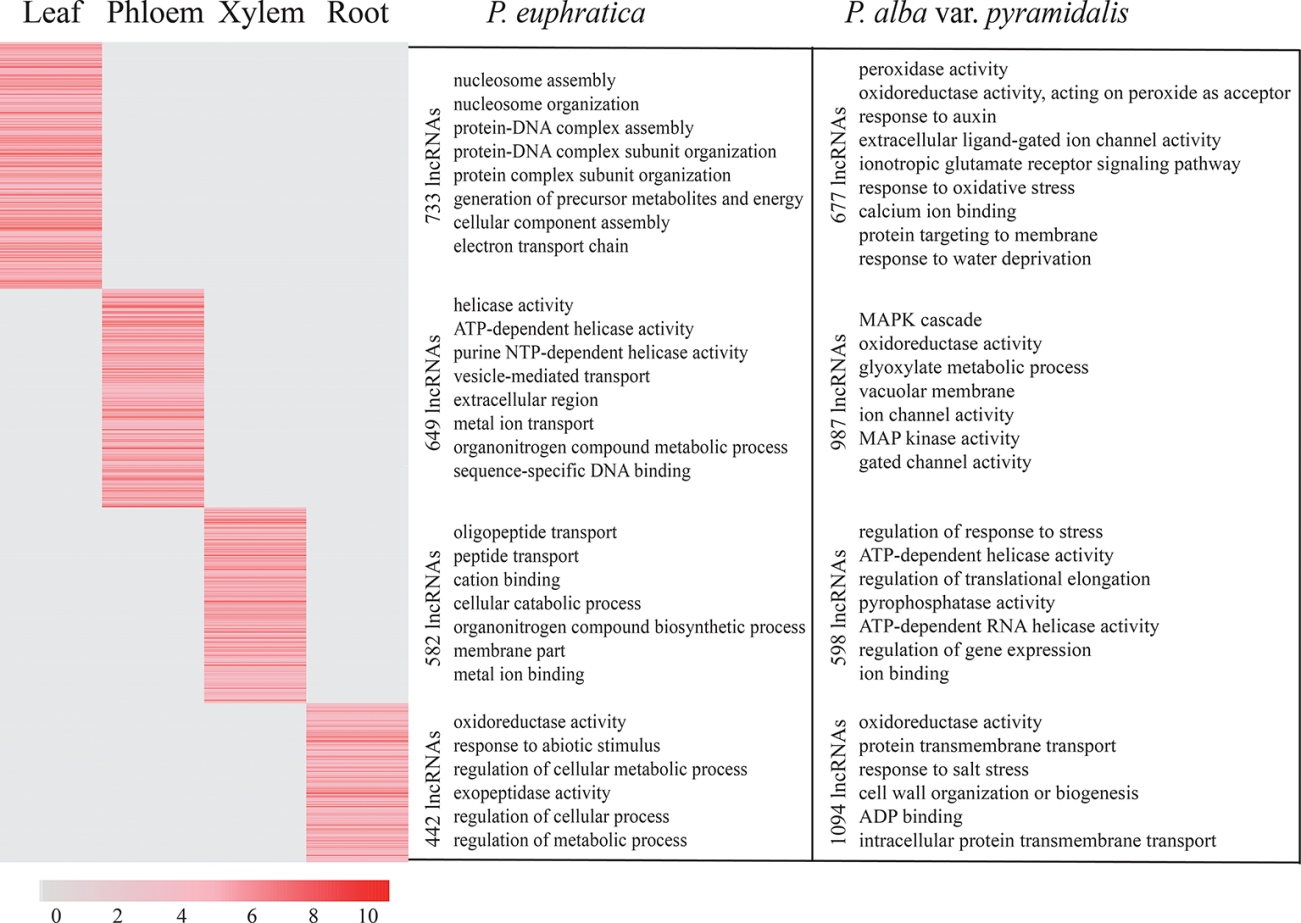
Tissue-specific DE lncRNAs and their enriched functions in the four tissue types of the two species of poplar.

Compared with mRNAs, most DE lncRNAs showed high tissue specificity, and only 153 and 80 of them were found to be differentially expressed between all four tissue types in the two poplars, respectively. We further identified 2,406 lncRNAs in *P. euphratica* and 3,356 lncRNAs in *P. alba* var. *pyramidalis* to be tissue-specific DE lncRNAs (**Supplementary Data S11–12**). These tissue-specific DE lncRNA numbers and functions also varied greatly between the four tissue types and between the two species of poplar (**Figure 6**). In *P. euphratica*, we found that the functions of “oxidoreductase activity” were only enriched in the root and that other tissues were mainly enriched in “ion transport.” However, in *P. alba* var. *pyramidalis*, the functions of “oxidoreductase activity” and “salt response” could be found in almost all tissues, except for the xylem. The enriched functions of these lncRNAs in the different tissue types indicated that different tissue types have evolved different salt stress response mechanisms and that the salt stress response mechanisms in the two poplars are varied.

### Comparison of lncRNAs in the Two Poplars

**Figure 7:**
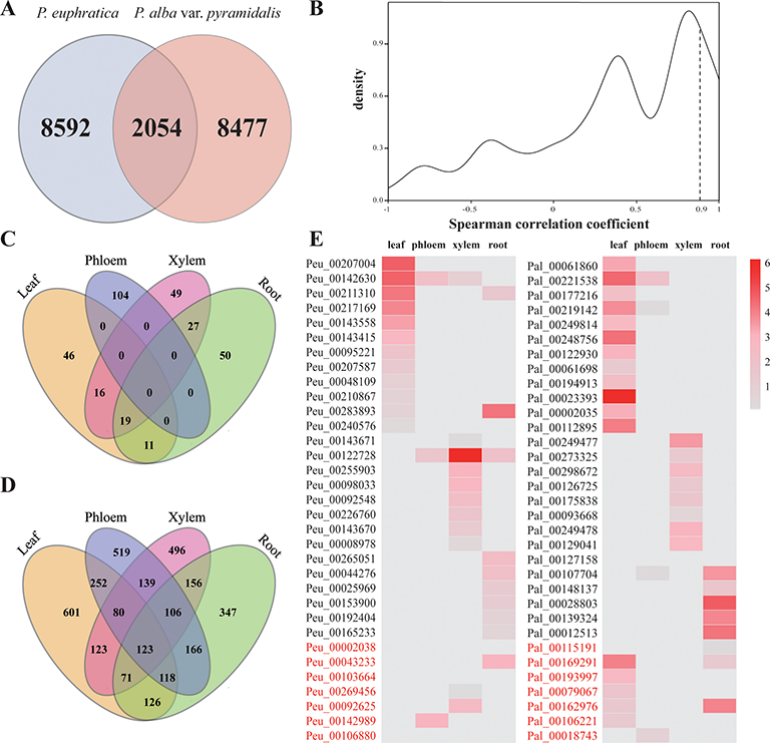
Comparison analysis of lncRNAs in the two species of poplar. **(A)** Homologous lncRNAs in two poplars. **(B)** Spearman correlation coefficient of homologous lncRNAs. **(C)** Distribution of lncRNAs with high expression level in *P. euphratica* but not in *P. alba* var. *pyramidalis* in four tissues. **(D)** Distribution of species-specific DE lncRNAs in *P. euphratica* in four tissues. **(E)** The expression patterns of homologous tissue-specific expressed lncRNAs. lncRNAs with red color indicate homologous lncRNAs expressed in different tissues.

Although the numbers of lncRNAs identified in the two sister poplars do not have remarkable differences, only 2,054 lncRNAs (nearly 20% of all identified lncRNAs) were identified as being homologous to each other, and the remaining lncRNAs (over 80% of the lncRNAs) were specific to each poplar species (**Figure 7A**). We further explored the expression patterns of homologous lncRNAs by using the *Spearman* correlation coefficient. Only 293 pairs showed similar expression patterns in the four tissue types, and these pairs showed a highly specific expression pattern of lncRNAs between the two poplars (**Figure 7B**, **Supplementary Data S13**). The results indicated that the lncRNAs diverged greatly, even between closely related species.

Among these identified homologous lncRNA pairs, 322 pairs were identified to be highly expressed in *P. euphratica* compared with *P. alba* var. *pyramidalis* (FC > 4), including 92 pairs in leaf, 104 pairs in phloem, 111 pairs in xylem, and 107 pairs in root (**Figure 7C**, **Supplementary Data S14**). Functional analysis implied these lncRNAs might be involved in regulating the expression of salt-responsive genes, such as cystatin B, annexin 5, and calcium-dependent protein kinase 32 (*CPK32*). There were 8,592 lncRNAs identified to be specifically expressed in *P. euphratica* with 3,425 lncRNAs showed different expressed under salt stress (**Supplementary Data S15**). For these 3,425 lncRNAs, more than half showed tissue-specific differently expressed (**Figure 7D**) and were predicted to regulate the expression of salt-responsive genes, such as *osmotin 34*, *NHX7*, *RARE-COLD-INDUCIBLE 2B*, and *WRKY 33*. Functional enrichment analysis also implied these lncRNAs involved in “regulation of hydrolase activity” and “response to oxidative stress,” which might contribute to the salt tolerance of *P. euphratica*.

We then compared these tissue-specific lncRNAs with the two poplars, and 33 lncRNA pairs were found to be homologous. However, only 20 of them were found to be expressed in the same tissue, whereas the remaining 11 lncRNAs might have had diversified expression patterns between the two species of poplar (**Figure 7E**). We further identified 33 and 19 housekeeping lncRNAs in *P. euphratica* and *P. alba* var. *pyramidalis*, respectively, by filtering using a tissue-specific index of <0.1. The expression patterns of these lncRNAs were not regulated by salt stress and appeared to be consistent among the four tissue types (**Supplementary Data S16**).

Among the DE lncRNAs, 522 lncRNAs were identified as being homologous in the two species of poplar. About 24% of the PTGs of these homolog lncRNAs were also found to be homologous. In addition, only 5% of the lncRNA-target pairs showed similar expression patterns (*Spearman* test > 0.9). Similar expression patterns usually indicate similar functions. We further investigated the expression patterns under different salt concentrations in the four tissue types. We set four patterns for this analysis. In *P. euphratica*, we found that the leaf and root showed similar expression patterns, with most genes being slightly downregulated at a concentration of 150 mM NaCl and being obviously upregulated at 300 mM NaCl. The phloem and xylem showed similar patterns with most genes being upregulated with both 150 and 300 mM NaCl (**Supplementary Figure S5**). These results indicate the presence of similar salt response mechanisms between the leaf and root and the phloem and xylem. In *P. alba* var. *pyramidalis*, the expression patterns varied among the four tissue types, which differed from *P. euphratica* (**Supplementary Figure S5**), indicating a totally different salt response level or mechanism in the two poplars.

We then investigated the similarity of tissue-specific DE lncRNAs between the two poplars. One hundred forty-eight lncRNAs were found to be homologous among the four tissue types, indicating that the most tissue-specific DE lncRNAs were not homologous between the two species of poplars. For instance, we found that one lncRNA, *Pal_00132209*, which was similar to a salt response lncRNA *DRIR* in *Arabidopsis* (Qin et al., 2017), showed differential expression in the xylem of *P. alba* var. *pyramidalis* (**Supplementary Figure S3C**), whereas no similar lncRNAs were found in *P. euphratica*. This lncRNA located approximately 1 kb upstream of a target kinase protein gene, namely, *PAYT016969.1*. The tissue-specific responses to salt stress from lncRNA might greatly contribute to the accurate and complex response mechanisms in plants. All these results indicated the tissue and species-specific response to salt stress of lncRNAs.

## Discussion

*P. euphratica* and *P. alba* var. *pyramidalis* are two closely related poplars and have recently diversified in phylogeny. However, *P. alba* var. *pyramidalis* exhibits a salt sensitive phenotype, whereas *P. euphratica* displays a high tolerance to salt. Comparison analysis between the two closely related species would help to explain the genetic mechanisms of their differentiation. In the study, we identified and characterized lncRNAs from four tissue types in the two species of poplar and explored their roles in the salt response. Our results demonstrated that lncRNAs exhibited weak evolutionary conservation in sequence, type, expression patterns, and regulatory models between the two closely related poplar species, which would provide flexible and different regulatory mechanisms to salt stress.

To date, a number of lncRNAs have been identified in various plants, it is difficult to reveal the functions of lncRNAs because most of the lncRNAs are weakly conserved and are expressed at low levels (Marques and Ponting, 2009; Ulitsky et al., 2011). In this study, lncRNAs showed low levels of similarity in length, expression, exon number, and relationship among the four tissue types compared with mRNAs. The comparison of lncRNAs between the two species of poplar showed a very low conservation of sequence, with only 20% of the lncRNAs found to be homologous. However, the percentage was higher than that compared with other species, such as *Arabidopsis* and rice (Wang et al., 2014a). These lncRNAs with similar *cis*-function across species may have conserved synteny with their target genes (Ulitsky et al., 2011, Herzog et al., 2014). Additionally, only 14% of these homologous lncRNAs were found to have similar expression patterns in all four tissue types. Finally, the PTGs of homolog lncRNAs were also found to have low similarity (only 24% were found to be homologous), and the regulation of lncRNAs to PTGs was very flexible. These results indicated that lncRNAs have a great variety not only in sequence similarity but also in expression patterns and target gene regulation, which would result in the diverse regulatory functions of lncRNAs in different species.

LncRNAs showed varied expression patterns in the two poplars. In *P. euphratica*, the leaf and root showed similar expression patterns, and phloem and xylem showed similar expression patterns. However, in *P. alba* var. *pyramidalis*, the expression patterns varied among the four tissue types and were different from those observed in *P. euphratica*. The highly tissue specific and induced by numerous biotic and/or abiotic stressors of lncRNAs would contribute toward improving the tolerance of the plant to various stressors (Liu et al., 2015b). The significant differences in lncRNAs expression patterns between the closely related species also suggest their rapid evolution (Marques and Ponting 2009; Ulitsky et al., 2011), which may contribute to the diversity among species, such as the differences in tolerance to salt stress between *P. euphratica* and *P. alba* var. *pyramidalis*. In *P. euphratica*, the contents of Na^+^ in the leaf and root did not change significantly, whereas the Na^+^ content of *P. alba* var. *pyramidalis* changed greatly, which indicated that *P. euphratica* could transport excess ions between its tissues, such as the root and leaf, maintaining ionic homeostasis and providing developmental plasticity through lncRNA regulation (Bazin and Bailey-Serres, 2015).

Differential expression of tissue-specific lncRNAs is another feature that may allow them to execute their functions in a more flexible manner. In *P. alba* var. *pyramidalis*, lncRNAs were found to be involved in oxidoreductase activity in almost all of the tissue types, whereas in *P. euphratica*, lncRNAs involved in the regulation of oxidoreductase activity were only found in the root. These results were highly related to the expression patterns of the target genes, such as members of the HKT family (high-affinity K^+^ transporters), an expanded gene family in *P. euphratica* genome (Ma et al., 2013). Among them, *PeuHKT1;1* was expressed mainly in the root, whereas *PeuHKT1;3* was expressed mainly in the leaf. Interestingly, we found that a lncRNA (*Peu_00167161*) showed similar expression trend with *HKT1*; 1 in the leaf but showed opposite expression trend in the root. The divergence of duplicated HKT genes in the expression patterns is beneficial to *P. euphratica* either through the exclusion of Na^+^ by the root or through decreasing the accumulation of Na^+^ in the leaf and can further contribute to maintaining ion homeostasis (Maser et al., 2002; Byrt et al., 2007; Yu et al., 2017). However, in *P. alba* var. *pyramidalis*, *HKT1* did not show diverse expression patterns. *Pal_00184400* and its predicted target gene, *HKT1*, were primarily differentially expressed in xylem (**Supplementary Figure S3D**). These results showed that DE lncRNAs in different tissues can help plants respond to salt stress with different regulationary manners.

Some conserved elements related to lncRNAs may be associated with plant growth and the salt response (Zhang et al., 2014). In this study, we found that lncRNAs in both poplar species have conserved elements related to plant growth and showed similar or opposite expression trend with genes that play core roles in plant growth, such as *Aux/IAA*, *NAC3*, and *WRKY8*, to promote plant development. Transcription factors, *NAC3* and *WRKY8*, were also identified as responding to salt stress in *P. euphratica* and function in the drought or salt stress response (Nakashima et al., 2012; Hu et al., 2013; Qin et al., 2017). A lncRNA (*Pal_00132209*) was identified as a homolog of *DRIR* in *Arabidopsis*, which could improve the tolerance of the plant to salt stress by affecting the activity of fucosyltransferase or *NAC3* or by regulating the redox status (Qin et al., 2017). This result indicated that lncRNAs with conserved elements may regulate their target genes to further control plant growth and the response to salt stress.

In summary, we identified and characterized lncRNAs in two closely related poplars by using stand-specific RNA-seq methods. We found that lncRNAs in both sister poplars showed varied regulation styles in terms of target genes, expression with high tissue specificity, low evolutionary conservation, and low expression levels. However, conserved elements related to lncRNAs were also found in the two sister poplar species. Taken together, tissue-specific expression and the unconservative gene sequences of lncRNAs provide multiple strategies to improve tolerance to salt stress, whereas conserved elements of lncRNAs might be involved in regulating the important processes of plant growth and development.

## Author Contributions

DW supervised the project. JM, YF and XB analyzed and interpreted data. WL and XS participated in design and drafting of the manuscript. YF, QB, SS, and QL performed the experiments during this study. All authors read and approved the final manuscript.

## Funding

The research was supported by the National Science Foundation of China (31870580) and the Fundamental Research Funds for the Central Universities (lzujbky-2017-k14).

## Conflict of Interest Statement

The authors declare that the research was conducted in the absence of any commercial or financial relationships that coud be construed as a potential conflict of interest.
