# Supplementary material for: Genome-Wide Identification of Long Noncoding RNAs and Their Responses to Salt Stress in Two Closely Related Poplars
Source: Front Genet. 2019 Sep 5;10:777. doi: 10.3389/fgene.2019.00777 (PMC6739720; doi:10.3389/fgene.2019.00777)
Supplement: Supplementary file 2 [file Table_2.docx]

Supplementary Figure legends:

**Figure S1:** The identification process of lncRNAs.

**Figure S2:** Clustering results of mRNAs and lncRNAs in four tissues under different salt concentration in *P. euphratica* (A) and *P. alba* var. *pyramidalis* (B).

**Figure S3:** qRT-PCR (quantitative real-time PCR) verification of four selected different expressed lncNRAs. Comparison of RNA sequencing (RNA-Seq) data (blue bar) with qRT-PCR data (red line). The normalized expression levels (FPKM) from the RNA-Seq results are indicated on the y-axis to the left. The relative qRT-PCR expression level is shown on the y-axis to the right. 18S RNA was used as an internal control. Primers: 18S RNA, F GATTCTATGGGTGGTGGTGG, R CAGGCTGAGGTCTCGTTCG; Peu_00167161, F AGCAGTGACGTTGTTGATGGA, R GCCCACTCCGTATGTGTGAA; Pal_00041124, F ATGCATGATGCCACTCCCTT, R ACAGGGACCTAGCTTCCGTTA; Pal_00132209, F TGCAATTTCATGCAAGGGATACAA, R TCAGTTGGGAGAGCGTCAGA; Pal_00184400, F GGTGGCTTGCATGTTTGAGG, R CATGCTGCCAAGTGCAATCA.

**Figure S4:** The spearman correlation coefficient of lncRNA and its target genes in *P. euphratica* (A) and *P. alba* var. *pyramidalis* (B).

**Figure S5:** The expression patterns of lncRNAs in different salt concentration in four tissues in *P. euphratica* (A) and *P. alba* var. *pyramidalis* (B).


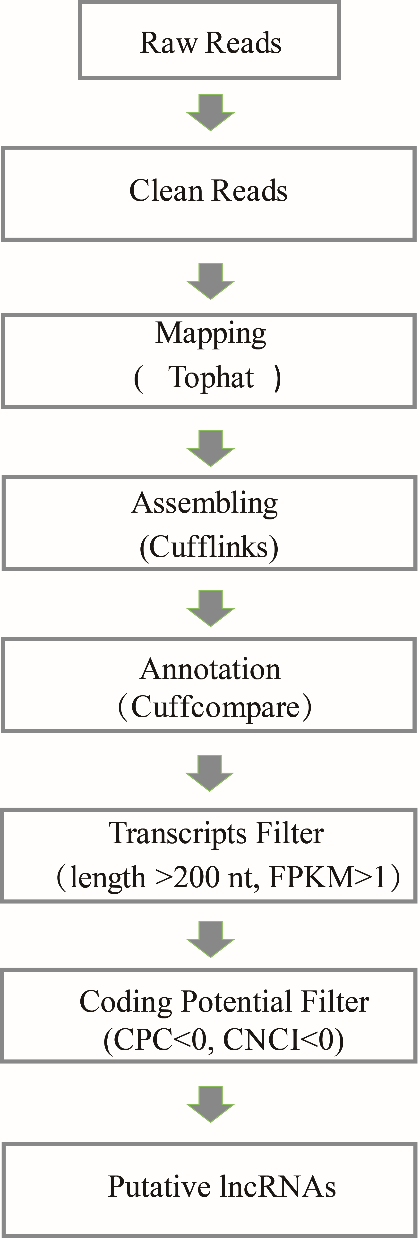


Figure S1: The identification process of lncRNAs.


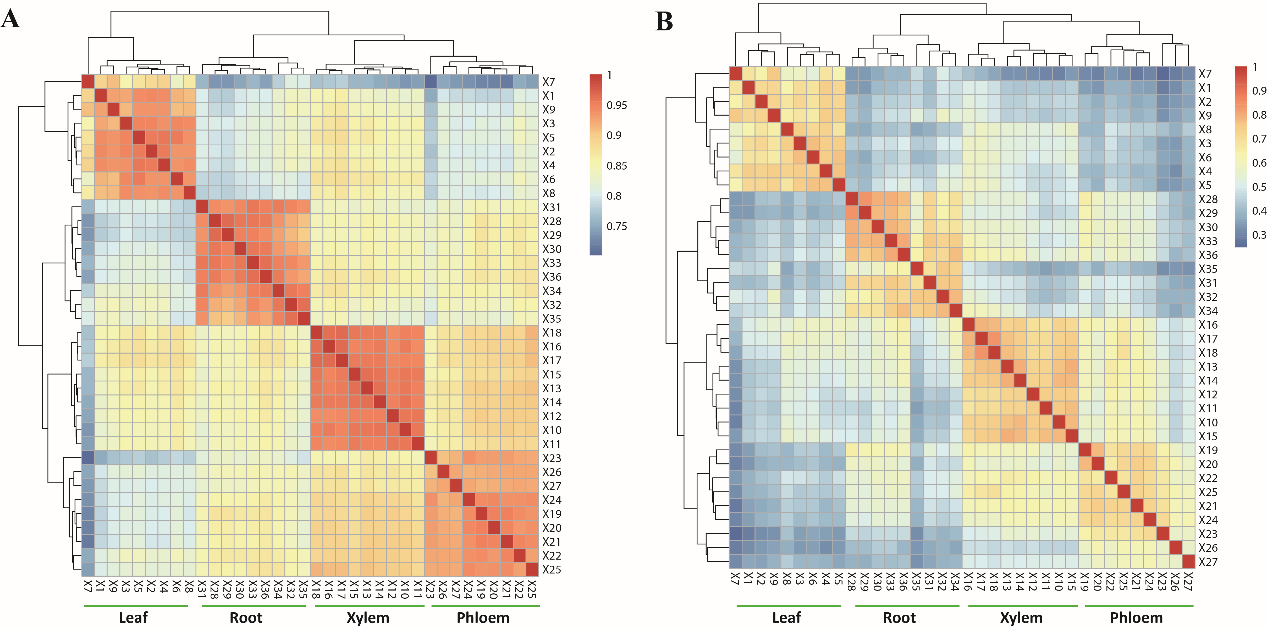


Figure S2: Clustering results of mRNAs and lncRNAs in four tissues under different salt concentration in *P. euphratica* (A) and *P. alba* var. *pyramidalis* (B).

**
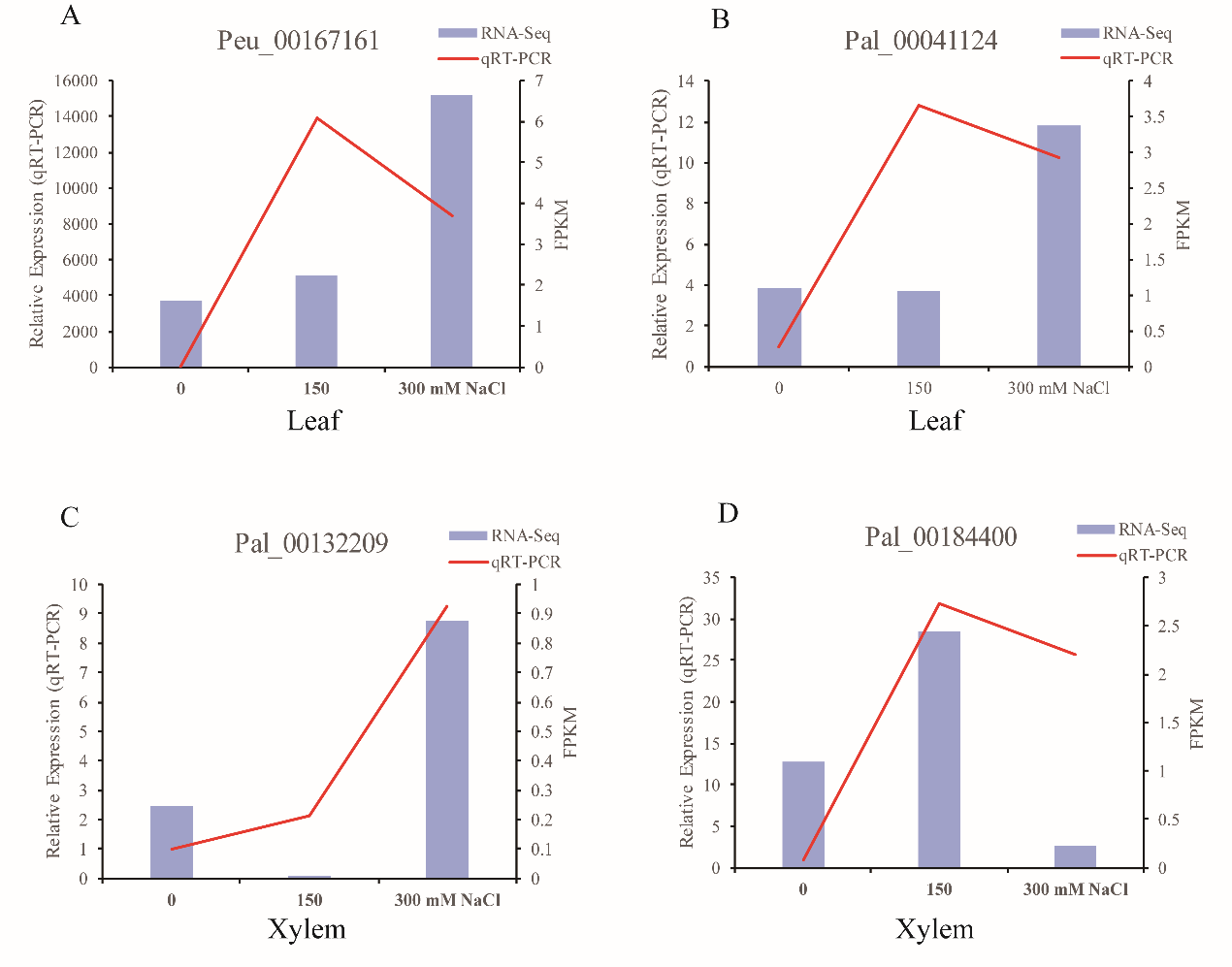
**

Figure S3: qRT-PCR (quantitative real-time PCR) verification of four selected different expressed lncNRAs. Comparison of RNA sequencing (RNA-Seq) data (blue bar) with qRT-PCR data (red line). The normalized expression levels (FPKM) from the RNA-Seq results are indicated on the y-axis to the left. The relative qRT-PCR expression level is shown on the y-axis to the right. 18S RNA was used as an internal control. Primers: 18S RNA, F GATTCTATGGGTGGTGGTGG, R CAGGCTGAGGTCTCGTTCG; Peu_00167161, F AGCAGTGACGTTGTTGATGGA, R GCCCACTCCGTATGTGTGAA; Pal_00041124, F ATGCATGATGCCACTCCCTT, R ACAGGGACCTAGCTTCCGTTA; Pal_00132209, F TGCAATTTCATGCAAGGGATACAA, R TCAGTTGGGAGAGCGTCAGA; Pal_00184400, F GGTGGCTTGCATGTTTGAGG, R CATGCTGCCAAGTGCAATCA.


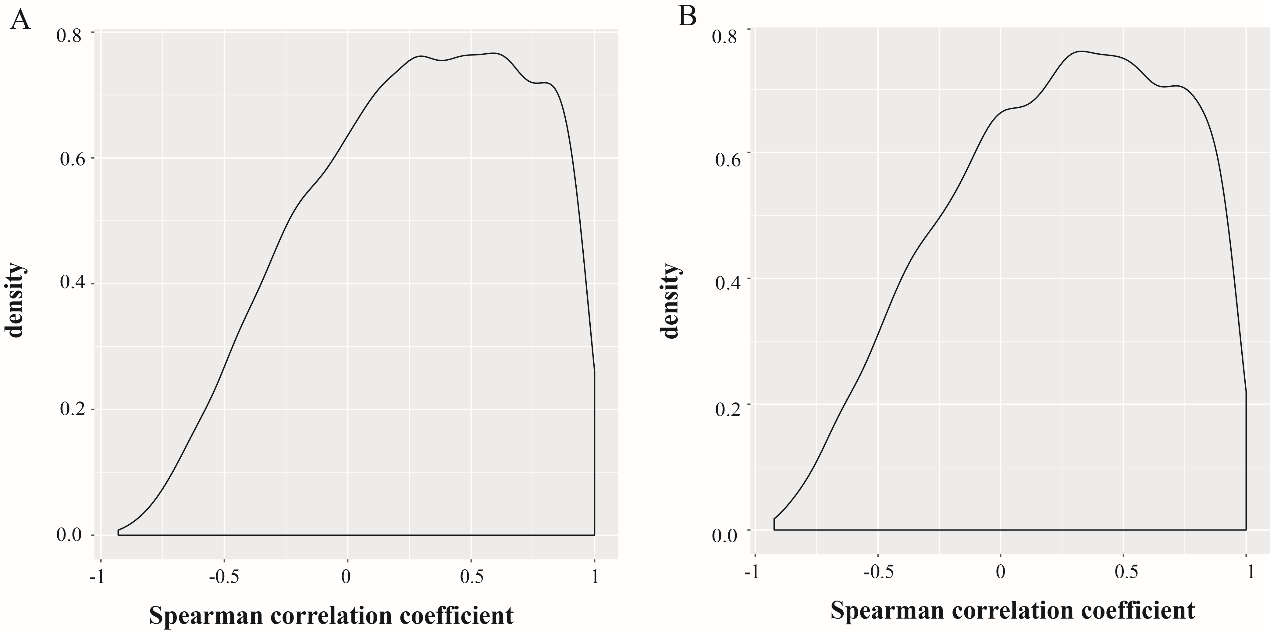


Figure S4: The spearman correlation coefficient of lncRNA and its target genes in *P. euphratica* (A) and *P. alba* var. *pyramidalis* (B).


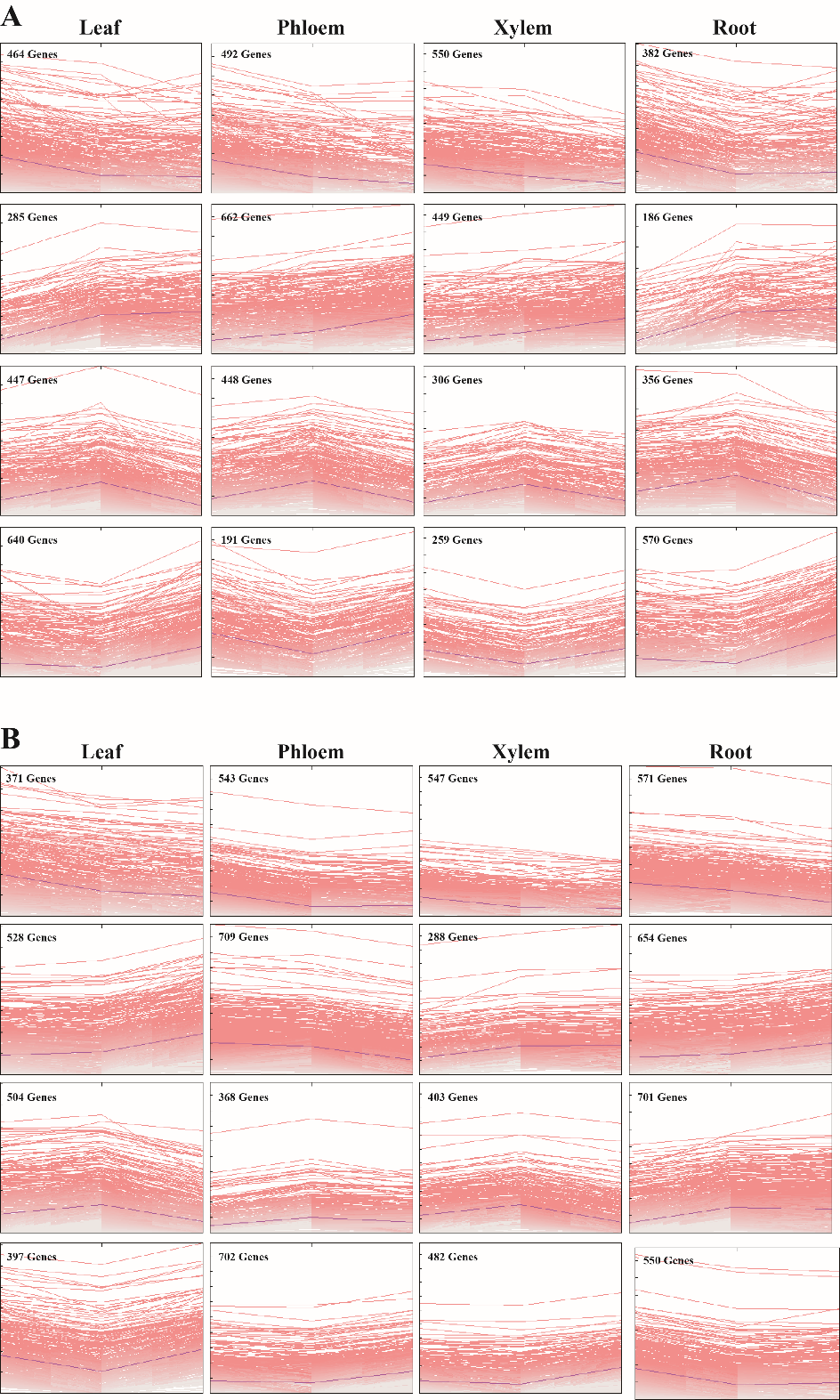


Figure S5: The expression patterns of lncRNAs in different salt concentration in four tissues in *P. euphratica* (A) and *P. alba* var. *pyramidalis* (B).
